# Heart rate variability as a dual-use digital biomarker: integrating clinical, AI, and operational perspectives on human performance and resilience

**DOI:** 10.1186/s12872-026-05543-z

**Published:** 2026-01-24

**Authors:** Alexandru Burlacu, Crischentian Brinza, Oana Geman, Matti Karppa, D. Jude Hemanth

**Affiliations:** 1 Faculty of Medicine, University of Medicine and Pharmacy “Grigore T. Popa”, Iasi, 700115 Romania; 2 Institute of Cardiovascular Diseases “Prof. Dr. George I.M. Georgescu”, Iasi, 700503 Romania; 3 Division of Data Science and Artificial Intelligence, Computer Science and Engineering, Chalmers University of Technology, and Gothenburg University, Gothenburg, Sweden; 4 Department of Electronics and Communication Engineering, Karunya Institute of Technology and Sciences, Coimbatore, India

**Keywords:** Heart rate variability, Autonomic nervous system, Operational medicine, AI, Dual-use, Prediction, Combat environments

## Abstract

**Background:**

Heart rate variability (HRV) reflects autonomic regulation and has emerged as a dual-use digital biomarker across clinical care and operational performance. We sought to integrate evidence on HRV’s physiological basis, clinical utility, defense applications, and AI-enabled analytics, and to propose a cross-sector framework for predictive, ethical deployment.

**Methods:**

We conducted a structured literature review in MEDLINE (PubMed), Embase, and Scopus between July 1st and August 31st, 2025, without language restriction. Eligible studies reported human HRV parameters measured in clinical, operational/defense, or AI contexts. Owing to heterogeneity, findings were summarized narratively across five domains: physiology, clinical applications, operational use, AI/predictive analytics, and ethics/standardization.

**Results:**

Evidence from military and operational studies supports HRV as a physiological indicator of stress accumulation, fatigue, and recovery during sustained workload and mission exposure. Across training environments, continuous HRV monitoring captured early autonomic changes preceding measurable performance decline or clinical symptoms. During prolonged field exercises, nocturnal HRV reductions consistently reflected accumulated allostatic load, while daily fluctuations in SDNN, RMSSD, and LF/HF ratios revealed real-time adaptations to physical exertion, sleep deprivation, and psychological strain. These dynamic shifts offered a quantifiable index of resilience, distinguishing between individuals able to sustain operational effectiveness and those approaching physiological or cognitive exhaustion. AI further enhances this capability by identifying non-linear and context-dependent HRV patterns that precede fatigue or decompensation. Machine-learning models trained on multimodal data streams enable early detection of autonomic instability and predictive risk stratification in both training and operational theaters.

**Conclusions:**

HRV is not just a number—it is a real-time window into how our bodies respond to life’s challenges, from the doctor’s office to the most demanding missions. What makes HRV so unique is its “dual-use” quality: it matters just as much for medical professionals caring for patients as it does for those monitoring the wellbeing and performance of people working under stress, such as soldiers or first responders. By treating HRV as a dual-use tool, one can bridge the worlds of healthcare and operational performance. This means the same heartbeat data that helps predict heart problems for a patient can also warn a team leader when their crew might be on the edge of exhaustion. But making the most of HRV in both settings requires to collect data consistently, analyze it with trustworthy AI, protect privacy, and put clear guidelines in place. In doing so, HRV becomes more than a monitor—a practical, ethical way to support better decisions, whether saving lives in a hospital or keeping people safe and effective under pressure.

## Introduction

Heart rate variability (HRV) represents the physiological fluctuation in the time intervals between consecutive cardiac cycles, reflecting the dynamic interaction between sympathetic and parasympathetic components of the autonomic nervous system. The capacity of HRV to quantify autonomic balance has made it one of the most informative non-invasive biomarkers of cardiovascular and neurophysiological modulation [1].

Low HRV has been consistently associated with increased cardiovascular and all-cause mortality in post-myocardial infarction populations and in patients with chronic cardiovascular disease [2, 3]. Beyond its prognostic role in cardiology, HRV could also evaluate the adaptive response to physical, cognitive, and emotional stress, thereby bridging the physiological and psychological dimensions of human performance [4].

Interest in HRV has expanded from the cardiac domain toward a broader interpretation of autonomic regulation as a universal indicator of resilience and system adaptability. Numerous studies have shown that reduced HRV has been associated with chronic stress, anxiety, burnout, and posttraumatic stress disorder (PTSD) [5, 6]. Conversely, higher HRV correlates with emotional flexibility, better stress recovery, and improved cognitive control [7]. Such findings have positioned HRV as a potential biomarker of both health and performance, aligning with the emerging concept of human performance optimization in operational medicine and defense research [8, 9].

Advances in sensor technology and wearable monitoring have further transformed HRV from a research parameter into a real-time physiological signal applicable across clinical and field environments. Continuous recordings obtained through portable chest straps or wrist sensors allow long-term assessment of autonomic responses during physical exertion, sleep, and recovery [10, 11]. Parallel progress in artificial intelligence (AI) and machine learning (ML) has enabled automated HRV analysis for detecting fatigue, stress overload, and early signs of cardiovascular instability [12]. These tools have been integrated into experimental frameworks within the U.S. Air Force Research Laboratory and NATO Science & Technology Organization (STO) programs to enhance situational awareness and adaptive workload management [13].

The concept of HRV as a dual-use digital biomarker emerges at this intersection between medicine and operational physiology. Dual-use implies simultaneous relevance for health monitoring and for optimizing human performance in high-stress or mission-critical settings. HRV provides a quantifiable link between clinical biomarkers of autonomic function and operational indicators of resilience, allowing the same physiological parameter to inform both preventive cardiology and defense readiness. While clinical studies validate HRV as a predictor of morbidity and mortality, defense-related investigations demonstrate its capacity to anticipate fatigue, cognitive decline, or physiological collapse in the field [14, 15].

In the present article, dual-use refers to the applicability of HRV across clinical health care and military operational performance contexts. This duality reflects the shared physiological foundations of autonomic regulation that inform patient care, resilience, readiness, and sustained performance under high-stress conditions.

Despite its growing adoption, current literature remains fragmented across disciplinary boundaries. Most medical publications focus on the prognostic or therapeutic implications of HRV, whereas military and AI-based research examine its use for workload and stress modeling without a clinical context. No integrative synthesis has yet framed HRV within a trans-sector perspective that unites these domains under a common physiological framework.

**Fig. 1:**
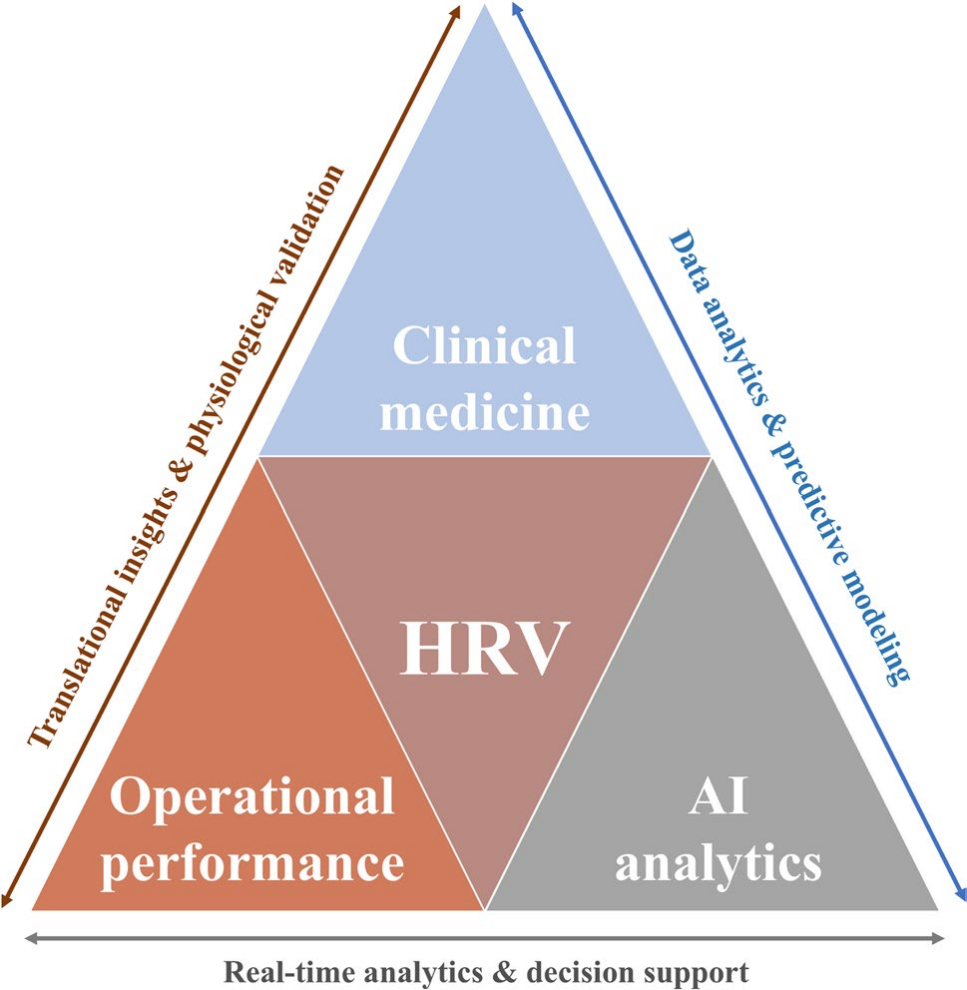
Conceptual framework of HRV integration across health and performance ecosystems

The present article aims to fill this gap by exploring HRV as a dual-use digital biomarker (Fig. 1), reviewing its physiological foundations, established clinical applications, and emerging roles in AI-driven analytics, defense, and human performance monitoring. By connecting clinical and operational evidence, this analysis proposes a unified interpretation of HRV as a scalable, predictive, and ethically sensitive metric of human resilience.

## Materials and methods

A structured literature review was performed to identify studies addressing clinical, operational, and artificial intelligence–based applications of HRV. The search was conducted in the MEDLINE (PubMed), Embase, and Scopus databases between July 1st and August 31st, 2025, and included all studies published up to that date, without language restriction. The search strategy included keywords related to HRV, stress, resilience, and military physiology. The main keywords combined in Boolean logic were “heart rate variability”, “stress”, “resilience”, “military”, “defense”, “operational medicine”, “combat”, “human performance”, and “tactical”. In addition, technical reports and scientific documents from NATO STO, the U.S. Department of Defense Human Performance Optimization (HPO) program, and the European Defense Agency (EDA) were reviewed to include data relevant to HRV applications in operational and defense research.

**Fig. 2:**
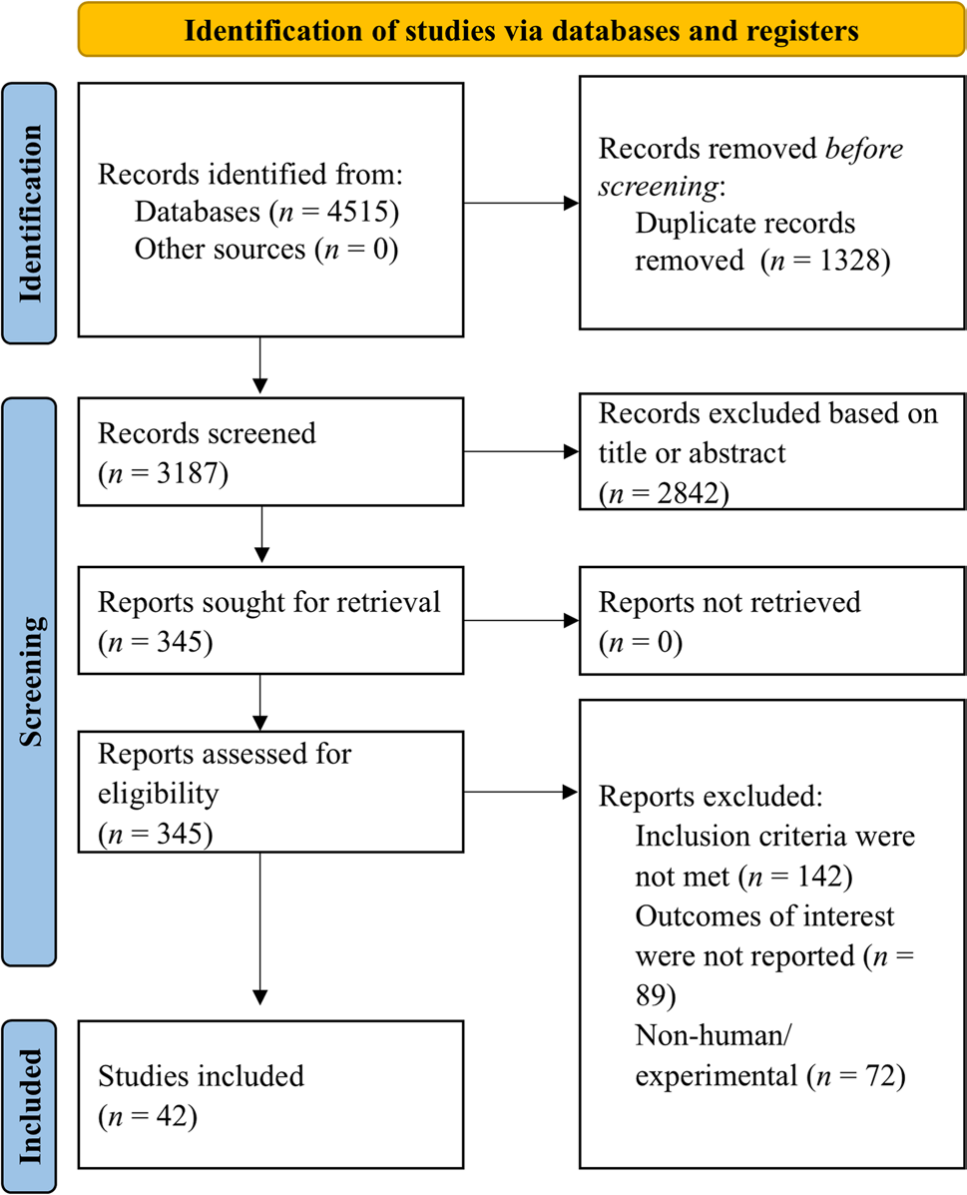
Prisma flow-chart of search process

Studies were considered eligible if they reported original human data evaluating HRV in a clinical, AI, or operational context. The inclusion criteria comprised the investigation of standard HRV indices, whether in the time-domain, frequency-domain, or non-linear domain. Studies focusing on disease prediction, stress physiology, or rehabilitation, and operational research studies describing HRV in a military context were included. Exclusion criteria comprised animal studies without translational relevance, conference abstracts, letters, commentaries, or experimental reports lacking standardized HRV measurement (Fig. 2).

After the database search, two investigators independently reviewed the titles and abstracts, while full-text evaluation was subsequently performed to confirm eligibility. Disagreements were resolved by consensus, and in case of persistent divergence, a third reviewer was consulted. The results were synthesized narratively, as substantial methodological heterogeneity prevented formal quantitative pooling.

Given the multidisciplinary scope of this analysis, encompassing cardiology, psychology, defense physiology, and computational sciences, results were integrated into a narrative synthesis organized along five major domains: physiological foundations, clinical applications, operational and defense implications, AI-based analytics, and ethical or standardization challenges. Data were summarized using descriptive statistics when comparable metrics were available.

This article presents a structured narrative review with a narrative synthesis. Therefore, a formal risk-of-bias assessment (as typically performed in systematic reviews) was not conducted.

## Physiological foundations of HRV

HRV constitutes physiological oscillation in the intervals between consecutive cardiac cycles, reflecting the dynamic interaction between sympathetic and parasympathetic components of the autonomic nervous system. Therefore, HRV reflects the capacity of the cardiovascular system to maintain homeostasis through flexible modulation of autonomic input [16]. Under normal conditions, parasympathetic influence predominates at rest, promoting an increase in HRV parameters, whereas sympathetic activation during stress, exercise, or disease decreases variability. Consequently, short-term HRV can evaluate the balance between these two components, referred to as sympathovagal balance, and serves as a non-invasive marker for central autonomic regulation [1, 4].

Quantitatively, HRV can be analyzed across three principal domains. Time-domain parameters are derived from the direct measurement of RR intervals obtained from electrocardiographic recordings [17]. Frequency-domain analysis, obtained through spectral decomposition, separates the signal into discrete frequency bands: the high-frequency (HF) component, which corresponds mainly to vagal modulation, and the low-frequency (LF) component, reflecting combined sympathetic and parasympathetic influences mediated by baroreflex mechanisms. The LF/HF ratio is often used as an indirect marker of sympathovagal balance, though its interpretation requires caution, especially under non-stationary conditions [18].

Beyond linear approaches, non-linear methods capture the complex, fractal nature of cardiovascular control. These methods highlight that healthy physiological systems exhibit organized complexity rather than randomness, an attribute lost in autonomic dysfunction [19].

Although the LF/HF ratio has historically been reported as an index of sympathovagal balance, this interpretation is increasingly debated and should be regarded as context-dependent rather than a direct measure of sympathetic-to-parasympathetic activity. LF and HF components could be affected by respiration, posture, physical activity, and non-stationary dynamics, conditions that commonly characterize real-world operational monitoring. Consequently, LF/HF can be misleading when used as a standalone surrogate of autonomic balance, particularly during exercise, acute stress exposure, or ambulatory environments. Thus, a physiologically interpretation should emphasize multi-metric patterns (e.g., time-domain indices such as SDNN/RMSSD, spectral components considered with respiratory context, and non-linear measures), rigorous artifact control, and, especially in dual-use settings, baseline-relative changes and trajectories rather than absolute thresholds derived from heterogeneous cohorts [17, 18].

Multiple factors influence HRV in both physiological and pathological contexts. Age and sex modulate baseline values, with progressive vagal withdrawal occurring across the lifespan and generally higher HRV observed in females before menopause [20]. Physical conditioning enhances vagal tone and increases HRV, while sedentary lifestyle, obesity, and poor sleep decrease it [21].

Overall, HRV constitutes a dynamic interface between neurophysiology, cardiovascular regulation, and environmental adaptation. Understanding its physiological basis provides the foundation for interpreting its clinical, operational, and AI-driven applications.

## Clinical applications of HRV

The clinical relevance of heart rate variability (HRV) stems from its capacity to reflect the integrity of autonomic regulation and the adaptability of cardiovascular control mechanisms under physiological and pathological stress. Over the last three decades, HRV has evolved from a physiological curiosity into a validated biomarker with diagnostic, prognostic, and therapeutic implications across multiple medical disciplines. Reduced HRV consistently indicates autonomic imbalance, sympathetic predominance, and diminished vagal tone—all predictors of adverse cardiovascular and systemic outcomes [4].

### Cardiovascular disease and mortality prediction

The first robust clinical evidence linking HRV to patient outcomes emerged from studies of myocardial infarction (MI). In 1987, Kleiger and colleagues reported that patients with a standard deviation of normal-to-normal RR intervals (SDNN) below 50 ms had a significantly higher mortality risk at 4-year follow-up after acute myocardial infarction compared with those above 100 ms [2]. Subsequent analyses confirmed that depressed HRV predicts both all-cause and cardiac mortality, independent of left ventricular ejection fraction or arrhythmic burden [22]. Meta-analyses have reinforced these observations, in patients with coronary artery disease or heart failure, lower time- and frequency-domain indices were consistently associated with higher mortality and major adverse cardiac events [3, 23].

In contemporary MI cohorts, HRV retains prognostic value, although effect sizes vary with timing of measurement and revascularization strategies. A recent meta-analysis, which included 27 studies in post-MI patients, found that low SDNN showed the strongest association with cardiac mortality (RR 4.19; 95% CI: 3.36–5.22), while the HRV index best predicted total mortality (RR 3.60; 95% CI: 2.30–5.64) [24]. In STEMI patients who underwent primary percutaneous coronary intervention, pre-discharge 24-h HRV maintained prognostic relevance, as lower SDNN and LF were associated with a higher risk of major adverse clinical events [25].

HRV measurement also provides complementary prognostic information beyond conventional risk stratification tools such as left ventricular ejection fraction or clinical scoring systems. In chronic heart failure, low HRV correlates with disease severity, elevated plasma norepinephrine, and poor functional capacity [26–28]. Likewise, in hypertensive and diabetic populations, autonomic dysregulation identified through HRV precedes overt cardiovascular complications, underscoring its potential as an early biomarker of end-organ damage [29–33].

### Metabolic and endocrine disorders

Autonomic dysfunction is a well-established feature of diabetes mellitus, where HRV serves as a non-invasive marker of cardiac autonomic neuropathy. Reduced SDNN and RMSSD values have been shown to predict progression of neuropathy and increased cardiovascular mortality among diabetic patients [34]. A 2021 meta-analysis involving over 2,000 individuals on chronic hemodialysis found that low HRV, notably diminished SDNN and increased LF/HF ratio, independently predicted all-cause and cardiovascular mortality [10]. These findings link autonomic dysregulation to metabolic and renal pathways of cardiovascular injury.

Insulin resistance, obesity, and metabolic syndrome also correlate with reduced HRV, suggesting impaired vagal modulation and increased sympathetic tone as standard mechanisms underlying cardiometabolic risk. Lifestyle interventions such as weight reduction and aerobic training improve HRV in parallel with glycemic and lipid control, indicating partial reversibility of autonomic impairment [35].

### Stress and mental health

HRV provides a physiological bridge between emotional state and cardiovascular function, allowing quantification of stress reactivity and recovery. Lower HRV is consistently observed in individuals with chronic stress, generalized anxiety disorder, and major depressive disorder, indicating diminished parasympathetic restraint over limbic activation [5, 36]. In posttraumatic stress disorder (PTSD), patients exhibit persistently low high-frequency power and elevated LF/HF ratios, patterns associated with hyperarousal and impaired extinction of fear responses [6]. Conversely, higher baseline HRV correlates with better emotional regulation, cognitive flexibility, and prefrontal control of autonomic output [37].

HRV biofeedback interventions, which employ paced breathing at an individual’s resonance frequency to enhance vagal tone, have shown beneficial effects in reducing anxiety, depressive symptoms, and stress-related disorders. Multiple randomized controlled trials and meta-analyses have documented significant increases in RMSSD and HF components following biofeedback training, along with improvements in perceived stress and emotional well-being [16, 17]. Such findings emphasize HRV not only as a diagnostic marker of psychophysiological state but also as a modifiable therapeutic target [38, 39].

Overall, HRV integrates data from cardiovascular, metabolic, and neuropsychological systems, providing a unified measure of autonomic health. Its decline across diverse conditions, from myocardial infarction to chronic stress, illustrates a shared pathophysiological pathway linking reduced adaptability with disease vulnerability. As wearable and contactless monitoring technologies advance, HRV assessment is increasingly feasible in both hospital and ambulatory settings, offering clinicians a continuous and multidimensional index of patient resilience.

## Operational and defense applications of HRV

The operational use of HRV has expanded far beyond its original clinical framework, becoming a cornerstone metric in the assessment of human performance, resilience, and physiological readiness under stress. Within the military, aviation, and defense innovation contexts, HRV represents one of the most practical and ethically sensitive examples of a dual-use digital biomarker. This physiological signal is simultaneously relevant to healthcare and operational decision-making.

### HRV as a marker of stress and fatigue in the field

In operational environments, heart rate variability (HRV) is increasingly used to monitor stress, fatigue, and physiological readiness among soldiers exposed to sustained physical and psychological load. Portable sensors such as the Zephyr BioHarness and Equivital Eq. 02 allow continuous recording of HRV signals during field training or missions, providing early indicators of autonomic imbalance before overt exhaustion appears.

A key study, Monitoring Responses to Basic Military Training with Heart Rate Variability [40], evaluated 48 recruits over 12 weeks of basic training and showed that nocturnal HRV effectively tracked accumulated allostatic load, identifying overreaching and fatigue earlier than subjective measures. Similarly, a study examining Heart Rate Variability to Assess Combat Readiness found that military trainees during operational preparation consistently showed lower HRV values, which were associated with physical exhaustion and reduced readiness, confirming HRV as a practical physiological indicator of combat fatigue [41].

A more recent investigation, Overnight Heart Rate Variability Responses to Military Combat Engineers, demonstrated progressive reductions in HRV during periods of sustained workload and field stress, suggesting cumulative autonomic strain even in the absence of overt clinical symptoms [42].

Taken together, these studies suggest HRV is a non-invasive biomarker that can reflect stress and fatigue in military populations, with potential utility for integration into real-time monitoring and adaptive workload management systems.

### Selection and training for high-stress environments

HRV provides a non-invasive and sensitive indicator of autonomic adaptability, which makes it valuable in the selection and training of personnel exposed to high physical and psychological stress. Across military and law-enforcement populations, HRV has been used to identify individuals with superior stress tolerance, recovery capacity, and cognitive control under pressure [43].

A cross-sectional study of active-duty Special Forces and Public Order military personnel demonstrated that HRV effectively distinguished between operational and administrative roles, with lower HRV associated with higher physical strain and exposure to acute stressors [44]. These findings support HRV as a potential tool for occupational profiling and for monitoring recovery during intensive operational duty.

In aviation training, pre-training HRV has been explored as a predictive marker of long-term performance. A recent study conducted at the United States Air Force Academy showed that candidates with higher baseline parasympathetic tone exhibited better resilience and adaptability during pilot training [45]. Such evidence reinforces the value of HRV as a physiological complement to cognitive and psychometric assessments in selection decision-making.

Technical evidence from the U.S. Air Force Research Laboratory – 711th Human Performance Wing, as well as from the NATO Science & Technology Organization Human Factors & Medicine Panels (HFM-302, HFM-333), supports the use of HRV feedback in stress-exposure and recovery training. These programs have shown that HRV-guided recovery strategies improved endurance, emotional regulation, and cognitive control under operational stress. However, most data derive from pilot and internal validation studies rather than peer-reviewed trials [9, 46].

Complementary physiological monitoring in military aviation and flight simulation has consistently shown HRV reductions during take-off, combat maneuvering, and landing phases, reflecting transient sympathetic activation and validating HRV as a sensitive marker of acute performance stress [13, 47, 48].

Overall, HRV-based assessment in candidate selection and high-stress training environments provides a quantifiable physiological perspective on resilience, complementing traditional metrics of physical fitness, cognition, and psychological stability. Its integration into modern training frameworks marks a shift toward evidence-based human performance optimization in both military and civilian high-demand professions [48].

### HRV-based early warning systems

The ability of HRV to detect autonomic imbalance before overt physiological collapse has generated growing interest in its integration into early-warning and remote-monitoring systems for both clinical and operational use. Because HRV alterations precede measurable changes in heart rate, blood pressure, or oxygen saturation, they can serve as anticipatory markers of physiological decompensation under stress, hypovolemia, or sepsis [49–51]. This temporal advantage makes HRV uniquely suited for early detection of stress overload or impending cardiovascular collapse in austere and combat environments.

Experimental work using simulated hemorrhage by lower-body negative pressure (LBNP) has shown that HRV indices decline well before the onset of hypotension. In tolerant versus non-tolerant subjects, high-frequency power and RMSSD decreased in proportion to central hypovolemia, confirming HRV as an early marker of cardiovascular decompensation [12]. These laboratory findings form the physiological foundation for HRV-based alert algorithms under development for military telemedicine.

In critical-care medicine, the same principle has been demonstrated clinically. Papaioannou et al. reported that reduced HRV predicted the onset of septic shock and multiple-organ failure hours before standard hemodynamic parameters changed, emphasizing its predictive diagnostic value [52]. Similar observations have been replicated in trauma and emergency settings, where reduced HRV was corelated with mortality and need for intensive resuscitation [49, 51].

Operational prototypes now exploit these insights. Wearable telemetry systems, such as the Zephyr BioHarness and Equivital Eq. 02, are being evaluated within the NATO Science & Technology Organization (STO) Human Factors & Medicine Panels (HFM-302, HFM-333) for continuous field triage. Parallel initiatives—e.g., DARPA’s Battlefield Assisted Trauma Distributed Observation Network (BATDOK)—combine HRV with accelerometry, temperature, and SpO₂ to create AI-enabled early-warning models capable of predicting physiological instability minutes before clinical recognition [46, 53].

Together, these converging lines of evidence position HRV as a key component of next-generation physiological surveillance, translating autonomic science into actionable, predictive intelligence for both battlefield medicine and civilian emergency response.

### Cognitive performance and decision fatigue

Heart rate variability (HRV) provides a sensitive physiological index of cognitive load, sustained attention, and mental fatigue, allowing quantification of autonomic cost during prolonged or high-demand decision-making. In operational environments—aviation, command and control, and cyber defense—where vigilance and situational awareness are critical, HRV serves as an objective correlate of cognitive strain and recovery capacity.

Early experimental evidence demonstrated that HRV dynamically tracks moment-to-moment changes in workload. Using simulated flight paradigms, De Rivecourt et al. (2008) found that reductions in high-frequency HRV and RMSSD paralleled increases in mental effort during take-off, navigation, and landing phases [13]. Similarly, Wilson (2002) analyzed psychophysiological responses of pilots. He demonstrated that low HRV and elevated LF/HF ratios were associated with high mental workload and degraded flight performance [47].

Extending to non-aviation contexts, Veltman and Gaillard (1998) reported progressive decreases in HRV with increasing task difficulty in cognitive-control simulations [48]. These results perceived HRV as a non-invasive proxy of executive effort across complex human–machine interactions.

Operational programs have echoed these findings. Technical assessments from the U.S. Air Force Research Laboratory – 711th Human Performance Wing and the NATO Science & Technology Organization Human Factors & Medicine Panels (HFM-302, HFM-333) highlight the potential of HRV-guided workload tracking to enhance situational awareness and mission performance [46].

Institutions such as the Defence Science and Technology Laboratory (DSTL, UK) and the Fraunhofer Institute for Communication, Information Processing and Ergonomics (FKIE, Germany) have conducted pilot programs on HRV-driven workload management for cockpit and mission-control environments. Developed within NATO STO and European Defence research frameworks, these initiatives explore adaptive human–machine interfaces that automatically adjust sensory load and task allocation in response to physiological stress signatures. While most results are presented in technical reports rather than peer-reviewed publications, they provide credible operational evidence that HRV can support real-time decision-making and cognitive-state monitoring [9].

Applied field studies have confirmed this relationship in tactical and security settings. In a systematic review, the authors observed that reduced HRV correlated with decision fatigue and attentional lapses during extended shifts [54]. Likewise, Luque-Casado et al. (2016) demonstrated that HRV declines progressively during sustained-attention tasks, reflecting the depletion of self-regulatory resources [55].

Taken together, these findings indicate that HRV can quantify the autonomic cost of cognitive control and support real-time workload management systems that optimize performance and prevent cognitive exhaustion. The convergence of experimental data and institutional initiatives suggests that HRV-based monitoring could become a core element of adaptive mission-support frameworks in both defense and civilian high-reliability operations.

### HRV in post-deployment recovery and resilience

Post-deployment and posttraumatic recovery represent critical stages in which autonomic flexibility, as reflected by HRV, plays a significant role in determining resilience and mental health outcomes. Reduced HRV has been consistently associated with posttraumatic stress disorder (PTSD), depression, and burnout, whereas restoration of parasympathetic modulation is linked to improved emotional regulation and recovery [56].

Early studies in combat veterans demonstrated markedly lower HRV compared with healthy controls, reflecting persistent sympathetic dominance and vagal withdrawal. Cohen et al. (1998) first reported significant HRV suppression in veterans with chronic PTSD [57]. More recent analyses using spectral HRV indices confirmed that decreased high-frequency power (HF) and elevated LF/HF ratios predict severity of re-experiencing and hyperarousal symptoms in PTSD populations [6, 15]. These findings position HRV as a physiological marker of impaired autonomic recovery and sustained threat reactivity after deployment.

Therapeutic interventions aiming to restore autonomic balance through HRV biofeedback have shown promise in improving both psychological and physiological resilience. Tan et al. (2011) implemented HRV-biofeedback training in active-duty service members. They observed significant increases in RMSSD and HF components alongside reductions in anxiety and PTSD symptom clusters [15]. In another study, higher baseline HRV predicted greater improvement during exposure-based psychotherapy for PTSD [58].

Beyond PTSD, reduced HRV has been linked to occupational burnout and emotional exhaustion among healthcare and emergency personnel exposed to chronic stress. Another study demonstrated that decreased HRV predicted later development of burnout symptoms in a longitudinal cohort [59]. Such data suggest that HRV serves not only as a post-trauma indicator but also as a preventive biomarker of cumulative stress dysregulation.

Institutional rehabilitation programs increasingly integrate HRV-guided interventions. The U.S. Department of Veterans Affairs (VA) and NATO Human Factors & Medicine Panel (HFM-326) have piloted HRV monitoring as part of resilience training and reintegration protocols for returning soldiers, employing wearable sensors to track autonomic recovery trajectories. Although much of this evidence remains in technical or pilot-report format, it underscores the practical feasibility of HRV monitoring for long-term psychophysiological rehabilitation [46].

The literature supports HRV as both a diagnostic and interventional biomarker in post-deployment care, linking autonomic flexibility with emotional resilience. Integration of HRV monitoring into multidisciplinary recovery programs may enhance individualized feedback, promote self-regulation, and objectively quantify progress toward physiological restoration.

### Implications for modern warfare and human performance optimization

Modern warfare increasingly depends on the capacity to sustain cognitive, emotional, and physiological performance under unpredictable and information-saturated conditions. As the boundaries between clinical health monitoring and operational readiness become increasingly blurred, heart rate variability (HRV) is emerging as a core biomarker for optimizing human performance, a central concept in both NATO and U.S. Department of Defense innovation frameworks [9].

The U.S. Department of Defense Human Performance Optimization (HPO) program, initially established within the Office of the Assistant Secretary of Defense for Health Affairs, identifies HRV as a key measure of autonomic readiness and recovery balance. HRV-based monitoring is now integrated into research pipelines at the U.S. Army Combat Capabilities Development Command (DEVCOM), the Air Force Research Laboratory, and the Naval Health Research Center, supporting adaptive workload management, fatigue mitigation, and predictive medical analytics. Such initiatives underscore the convergence of biomedical monitoring, data science, and operational ergonomics toward anticipatory, AI-enhanced performance management systems [46].

Internationally, the NATO Science & Technology Organization (STO) has articulated a similar strategic vision in its Human Systems and Behavior (HSB) and Human Factors & Medicine (HFM) panels, which explicitly recognize HRV as a dual-use physiological metric. The 2023 technical report STO-TR-HFM-333: Human Performance Optimization in Operational Environments identifies HRV as one of the most reliable and scalable indicators of resilience, fatigue, and workload regulation [9]. Likewise, European defense research programs coordinated by the European Defence Agency (EDA) and Fraunhofer Institutes (Germany) are incorporating HRV into human–AI teaming frameworks for pilot monitoring, cyber operations, and command center ergonomics [60].

Beyond defense applications, the same technology platforms are migrating into civilian sectors, aviation, healthcare, and emergency response, creating a feedback loop where operational physiology informs clinical innovation and vice versa. This dual flow reinforces HRV’s status as both a digital health metric and an operational readiness indicator, bridging public health and national security [61, 62].

While ethical and privacy challenges remain substantial, especially regarding continuous biometric surveillance, these cross-sector integrations demonstrate how HRV is evolving from a clinical research parameter into a strategic capability for monitoring, predicting, and sustaining human performance in complex systems. The shift from reactive medicine to predictive human performance physiology marks a fundamental transformation in how modern militaries conceptualize resilience and readiness [40].

### Methodological limitations of wearable-derived HRV

A key limitation of HRV monitoring in operational and field environments relates to the widespread use of photoplethysmography (PPG)-based wearable sensors rather than electrocardiography (ECG), which remains the clinical gold standard for HRV assessment. PPG-derived pulse interval series are inherently more susceptible to motion artifacts, variations in peripheral perfusion, skin temperature, and sensor-skin contact, all of which may impact signal quality during physical activity, vibration, or environmental stress. These factors can particularly affect short-term and vagally mediated HRV indices, such as RMSSD or non-linear metrics, leading to reduced reliability under dynamic conditions [63, 64].

Despite these limitations, recent validation studies provide encouraging evidence supporting the operational utility of selected wearable devices under controlled or low-motion conditions. A head-to-head comparison between smartwatch-derived PPG and high-resolution ECG in patients with cardiovascular disease demonstrated very high concordance for mean heart rate, SDANN, and very low-frequency power, while agreement was moderate for short-term variability indices. Similarly, validation of the Empatica E4 wristband showed comparable signal quality to Holter ECG recordings in the majority of cases under standardized conditions [63, 64].

An important consideration in the dual-use interpretation of HRV is the impact of pharmacological treatment in clinical populations. Many patients included in cardiovascular or neurological studies received medications that could influence autonomic regulation, including beta-blockers, antiarrhythmic drugs, and sedative agents. These therapies can substantially modify HRV indices, thus limiting direct comparison of absolute HRV values with those observed in generally healthy operational populations [65, 66].

Consequently, HRV metrics should not be interpreted as interchangeable across medicated clinical cohorts and unmedicated military or operational personnel. Within the proposed dual-use framework, the translational value of HRV lies not in cross-population equivalence of absolute values, but in its capacity to capture within-individual dynamics, including baseline-relative changes, recovery patterns, and deviations from expected physiological trajectories.

Another important aspect is that classical clinical prognostic studies have primarily relied on 24-hour ECG recordings of HRV, which capture circadian autonomic modulation and provide robust long-term risk stratification in cardiovascular disease. However, short-term HRV recordings, typically obtained over 5–10 min under standardized conditions, have also been extensively validated and shown to provide reliable and physiologically meaningful indices of autonomic regulation. Importantly, short-term HRV is particularly sensitive to acute autonomic fluctuations, capturing rapid responses to physical exertion, psychological stress, sleep deprivation, and environmental challenges. This property is critical in operational and military contexts, where the objective is not long-term prognosis but early detection of physiological strain, fatigue, or performance degradation. Within the dual-use framework proposed, long-term and short-term HRV should therefore be viewed as complementary tools. Long-term recordings inform on chronic risk pattern in clinical medicine, whereas short-term recordings enable real-time, context-aware monitoring of dynamic autonomic responses in high-stress operational environments [1, 22, 67].

Taken together, these findings indicate that while ECG remains essential for precise HRV quantification and clinical decision-making, PPG-based wearables can provide reliable and clinically meaningful HRV data when appropriately interpreted. In operational and military contexts, this supports the use of wearable-derived HRV as a complementary, trend-sensitive biomarker within secure and validated monitoring frameworks.

## AI-Driven and predictive analytics in HRV research

Building on the operational applications discussed above, contemporary advancements in artificial intelligence (AI) and machine learning (ML) are transforming HRV analysis from descriptive monitoring into predictive, actionable physiological insight. Through the integration of HRV with contextual, wearable, and multimodal data streams, it becomes feasible to anticipate autonomic dysregulation, fatigue, stress, and health deterioration, crucial considerations in both clinical care and high-performance operational settings [68].

### From classical signal analysis to AI-Augmented interpretation

Traditionally, HRV assessment has relied on time-domain (e.g., SDNN, RMSSD) and frequency-domain (LF/HF ratio) metrics, interpreted through linear models of autonomic nervous system (ANS) balance. However, these metrics may fail to capture subtle, transient, or non-linear patterns emerging under complex physiological stress. Studies employing ML/DL approaches show that HRV features fed into supervised models can better discriminate states of stress, load, and recovery. For example, a study using supervised learning algorithms on HRV data classified stress vs. relaxation states and emphasized the generalisability of ML models when trained on limited datasets [69, 70].

Another investigation demonstrated that models trained on HRV metrics from wearables could predict daily mental states in real-world conditions (students’ stress) via ML on HR/HRV sequences [71]. Such developments point toward HRV analysis evolving beyond static indices into dynamic, context-aware models embedded in wearable systems.

### Predictive models for health and operational readiness

The shift toward predictive modelling in HRV analytics is of particular relevance: by combining HRV time-series with contextual biosignals, ML algorithms can forecast risk, readiness, or impending physiological failure. For instance, studies have shown that HRV-based ML models can identify cardiovascular disease risk or heart failure status, using HRV features as input variables [72].

In the wearable-health domain, a machine-learning model applied to smartwatch data predicted HRV metrics and hence autonomic responses to environmental stressors (e.g., in heart-failure patients) with correlation *R* > 0.8 in some indices [64]. From an operational readiness perspective, such predictive capacity means HRV can serve not just as a snapshot biomarker, but as an early-warning indicator, enabling intervention (rest, redistribution of load) before performance degradation or health event occurs.

#### Signal quality and real-world data robustness

Real-world HRV acquisition, particularly in operational or high-mobility environments, is inherently challenged by signal loss, motion artifacts, intermittent sensor contact, and transmission noise. These factors represent a critical limitation of physiological monitoring outside controlled laboratory or clinical settings and directly affect the reliability of AI-based predictive analytics [61].

To address these constraints, contemporary AI-driven HRV pipelines typically incorporate Signal Quality Indices (SQI)and multi-stage data preprocessing frameworks prior to model inference. These steps commonly include beat-level artifact detection, ectopic beat correction, window-based SQI thresholding, and exclusion or down-weighting of low-confidence signal segments before feature extraction. Model outputs are generated only when predefined signal integrity criteria are met, thereby minimizing false-positive alerts driven by corrupted or incomplete data [73].

Beyond software-level mitigation, several advanced dual-use operational systems integrate hardware-level signal stabilization, secure capture, encryption, and robust data transmission architectures that substantially outperform consumer-grade wearable devices in terms of signal fidelity and continuity. While specific technical implementations cannot be disclosed due to security and confidentiality constraints, such systems demonstrate the feasibility of reliable HRV acquisition and AI-assisted analytics in demanding real-world contexts [19, 74].

Importantly, modern AI models increasingly rely on training strategies that explicitly account for noisy and partially incomplete datasets, including robust feature selection, ensemble learning, and uncertainty-aware inference. These approaches allow models to tolerate a degree of signal imperfection without generating excessive false positives. Nevertheless, signal quality assessment remains a prerequisite for meaningful interpretation, and AI-based HRV analytics should be regarded as decision-support tools operating within clearly defined data-quality boundaries rather than fully autonomous decision systems [68].

### Explainable AI and ethical dimensions

While powerful, AI/ML-based HRV analytics raise important questions of interpretability, trust, and ethics. Explainable AI (XAI) methods ensure that decision-making by ML models remains transparent and physiologically meaningful, essential in clinical or personnel-management contexts. A scoping review of HRV showed the rapid growth of ML/AI applications and emphasized the need for interpretable models and prospective validation [70].

Ethically, deploying HRV predictive systems in operational settings (e.g., military, high-demand workplaces) necessitates caution, as individual consent, data privacy, and the risk of misuse (e.g., biometric data for performance pressure) must be addressed. Moreover, model bias, generalizability across populations, and false alerts require governance frameworks tailored to human-performance ecosystems [75].

While deep learning approaches have demonstrated high performance in pattern recognition and prediction from HRV time series, their limited transparency remains a concern in clinical and operational environments where trust, accountability, and physiological interpretability are essential. In response, XAI methods are increasingly integrated into HRV analytics to mitigate the “black box” effect [68, 70].

In practice, XAI techniques, such as feature attribution, attention mechanisms, and post hoc explanation models, could be to identify which HRV features (e.g., SDNN, RMSSD, spectral components, or non-linear indices) contribute most strongly to a given prediction. Importantly, in both medical and military settings, AI-driven HRV systems are increasingly deployed within human-in-the-loop decision-support frameworks, where algorithmic outputs inform but do not replace clinical or operational judgment. This paradigm supports responsible use of AI in safety-critical contexts, ensuring that predictive analytics enhance situational awareness while maintaining transparency, oversight, and trust [68, 70, 75].

As HRV analytics continue to evolve, the integration of XAI could represent a necessary step toward ethically acceptable and operationally reliable AI-assisted monitoring systems, particularly in environments where wrong or non-interpretable decisions may carry significant clinical or mission-related consequences.

### Integrative HRV analytics: from biofeedback to autonomous systems

Integration of HRV analytics into adaptive systems represents the next frontier: AI-driven biofeedback loops that modulate interventions (breathing, recovery prompts, workload adjustments) based on live HRV state. For wearable sensors and digital health platforms, HRV forms part of a dynamic physiological feedback architecture. For example, studies show that wearable sensors + ML enable stress monitoring via HRV and real-time intervention suggestions [76].

In operational domains, this capability opens pathways toward human-machine system synchronization: HRV-informed changes in task load, UI complexity, or assistance level, thereby optimizing performance under stress while supporting recovery. The transition is from HRV as passive monitoring to HRV as an active control signal in autonomous systems [76].

### Outlook: toward a predictive human-performance ecosystem

The convergence of wearable HRV measurement, AI/ML modelling, and operational physiology heralds a predictive human-performance ecosystem, one in which physiological data continuously inform readiness, health status, and recovery. Future systems will likely incorporate multimodal data (ECG/PPG, HRV, respiration, motion, environmental context) into cloud-edge-device pipelines that support real-time analytics and adaptive intervention [77].

From a clinical standpoint, this means moving from reactive treatment toward proactive monitoring and intervention; in operational settings, it means sustained readiness, resilience, and prevention of mission-critical failure. To achieve this vision, technical, ethical, operational, and regulatory challenges (data standardization, sensor reliability, model validation, and workforce acceptance) must be addressed. When done right, AI-enhanced HRV analytics can bridge health optimisation and performance management, delivering value across medicine and high-stakes operations [78].

## Cross-sector and dual-use integrations

Building on the AI-driven predictive analytics discussed above, the accelerating convergence between clinical and operational physiology has positioned HRV as a truly **dual-use digital biomarker**, one bridging healthcare, defense, and technology. This section examines how HRV analytics are now shared, translated, and ethically governed across medical, occupational, and defense innovation ecosystems [79].

### Convergence between clinical and operational HRV ecosystems

The same HRV metrics used to quantify recovery in cardiology and stress-rehabilitation contexts are now being applied to assess fatigue, stress, and cognitive workload in pilots, soldiers, and first responders. Devices such as the Zephyr BioHarness, Equivital Eq. 02, and Empatica E4—initially validated in sports and rehabilitation medicine—have been adopted in NATO, U.S. Air Force, and European Defence Agency (EDA) programs for operational readiness monitoring [9, 80].

Both clinical and military research increasingly rely on comparable physiological frameworks. Short-term spectral indices (HF, LF/HF) and non-linear measures (sample entropy, Poincaré SD1/SD2) quantify autonomic flexibility and resilience in both patient recovery and high-stress missions. AI-driven analytics have transformed HRV from a retrospective biomarker into an anticipatory signal capable of identifying overload or fatigue hours before performance decline or clinical instability appears [81].

In addition, sex-related differences in autonomic regulation represent an important consideration in the interpretation of HRV across clinical and operational contexts. Baseline HRV values are influenced by biological sex, with generally higher vagal modulation observed in premenopausal females compared with age-matched males. Despite this established physiology, many operational and military HRV datasets remain male-dominated, reflecting historical recruitment patterns. As a result, validation of HRV-based predictive models and AI algorithms across sexes, particularly in female personnel remains limited. This represents a methodological gap within current dual-use frameworks and constitutes a direction for further research [20, 82].

Nevertheless, the dual-use value of HRV lies primarily in baseline-relative and trajectory-based interpretation rather than absolute cross-population comparisons. Such approaches partially mitigate sex-related differences but do not replace the need for dedicated sex-stratified validation. Future development of AI-enabled HRV analytics should therefore incorporate sex-aware modeling.

### Data interoperability and translational value

Modern HRV platforms are increasingly interoperable through standards such as HL7 FHIR and IEEE 11,073, enabling seamless integration of data from wearables with hospital information systems or command-center dashboards. This interoperability enables a continuous data pipeline between clinical monitoring and field operations [83].

Insights now flow bidirectionally. Algorithms initially trained on ICU datasets to detect sepsis and shock [84] have been adapted by defense research groups for predicting hypovolemia and fatigue under simulated combat stress [12]. Conversely, findings from NATO and U.S. Army resilience programs have informed HRV-guided recovery protocols for burnout prevention among healthcare workers [15].

NATO’s Human Factors & Medicine (HFM) panels describe this process as a “reverse transfer of resilience,” whereby operational physiology informs medical care, and clinical insight enhances mission performance.

### Applied scenarios: HRV-Driven decision support in the field

To illustrate the translational potential of HRV-based analytics across medical and operational domains, the following applied scenarios show how AI-enabled HRV monitoring can guide real-time decisions in high-risk environments:

1. Battlefield Triage and Autonomic Early Warning.
  Combat medics equipped with HRV telemetry in tactical vests (e.g., Zephyr BioHarness, Equivital EQ02) receive early alerts of autonomic collapse before measurable hypotension occurs. AI models trained on HRV + SpO₂ + motion data predict hemodynamic instability and color-code casualties for priority evacuation (“*amber alert*”) [87, 88]. The same algorithmic principles have proven effective in ICU sepsis prediction [14, 50].
2. Pilot and UAV Operator Cognitive-Load Management.
  During prolonged missions, reductions in HF power and RMSSD signal sympathetic over-activation. Adaptive cockpit software temporarily reduces display intensity or visual load, allowing for a brief parasympathetic recovery. HRV + EEG fusion models predict decision fatigue and mitigate performance errors [89, 90].
3. Special Operations Forces (SOF) Autonomic Readiness and Mission Endurance.
  Elite military units operate under sustained cognitive and physical stress, often with limited sleep, caloric restriction, and extreme environmental exposure. In such missions, autonomic resilience, the capacity to maintain physiological flexibility under load, has become a critical performance determinant. Continuous HRV monitoring provides individualized readiness profiles that reflect each operator’s recovery status and adaptive capacity.

The U.S. Army and NATO Human Factors & Medicine (HFM) panels have identified HRV as a core component of human-performance readiness monitoring, integrated with actigraphy, thermal strain estimation, and workload metrics to form composite “physiological readiness indices” [9, 46]. These models are being evaluated in special-operations training environments to detect early autonomic overstrain and to optimize rest-to-work ratios.

In pilot implementations, HRV deviations from baseline (e.g., sustained > 25% RMSSD decrease or LF/HF imbalance) are used by field physiologists to recommend micro-rest intervals, hydration, or mission-rotation adjustments—reducing cognitive errors and fatigue-related injuries. Preliminary findings from NATO SOF training trials suggest that such HRV-guided interventions enhance accuracy, reaction time, and situational awareness under prolonged operational load (NATO HFM-361 Pilot Study, 2024).

Beyond combat applications, similar HRV-based readiness analytics are now explored for civilian high-reliability teams such as surgical units, emergency-response crews, and air-traffic controllers, underscoring HRV’s translational value as a universal metric of resilience and sustained human performance [42].

## Post-deployment autonomic rehabilitation

Wearable HRV platforms track nightly vagal recovery in returning soldiers, providing personalized breathing or relaxation guidance. Longitudinal data feed into resilience databases managed by the U.S. Department of Veterans Affairs and NATO HFM-326 initiatives. Similar HRV-guided rehabilitation tools are deployed for PTSD and burnout prevention in healthcare professionals.

## Mass-casualty and disaster response

Drone-mounted sensors within DARPA’s BATDOK system extract HRV from remote photoplethysmography to categorize casualties (“stable – unstable – critical”) when direct vitals are inaccessible. The same AI architecture has been adapted for telemedicine triage in disaster and pandemic response (U.S. DoD BATDOK Project, 2022) [89].

Together, these examples highlight HRV’s unique capacity to serve as a shared physiological signal of survivability, integrating battlefield medicine, aerospace ergonomics, and civilian emergency care within a single predictive ecosystem.

### Ethical and governance harmonization

As HRV analytics cross boundaries between medicine and defense, ethical challenges emerge regarding data ownership, informed consent, and misuse. Dual-use systems must be governed by ethical interoperability, ensuring data collected for safety or health is not repurposed for coercive evaluation [75].

The European AI Act (2023), NATO Ethical AI for Human Performance (HFM-ET-203, 2023), and WHO Guidance on AI Ethics (2021) provide complementary governance frameworks emphasizing transparency, accountability, and human oversight. Defense programs are increasingly adopting medical-grade data protections, including de-identification, role-based access, and the precise separation of clinical and command analytics streams. Such measures maintain individual autonomy while preserving operational utility.

### Toward a unified human-performance continuum

As HRV platforms operate seamlessly across hospitals, laboratories, and operational theatres, the distinction between health monitoring and performance optimization is rapidly dissolving. AI-augmented HRV analytics now underpin a continuum of human-performance medicine, merging preventive cardiology, occupational physiology, and mission readiness within a shared ethical and scientific framework [90].

Future predictive ecosystems will integrate HRV with EEG, respiration, motion, and environmental sensors to enable closed-loop adaptation, balancing physiological resilience with operational efficiency. Within both civilian and defense infrastructures, HRV remains the most mature and evidence-based autonomic marker capable of uniting medical precision with mission reliability, an essential element in the evolution of predictive human-performance systems [91].

## Security, data Integrity, and standardization challenges

In modern operational medicine, the transmission and supervision of physiological data from the field must meet the same standards of accuracy and confidentiality as hospital-based monitoring. For HRV and related vital signs collected on the front line, the key principle is secure continuity of medical information, from the individual sensor to the physician supervising from the rear medical hub [92].

**Fig. 3:**
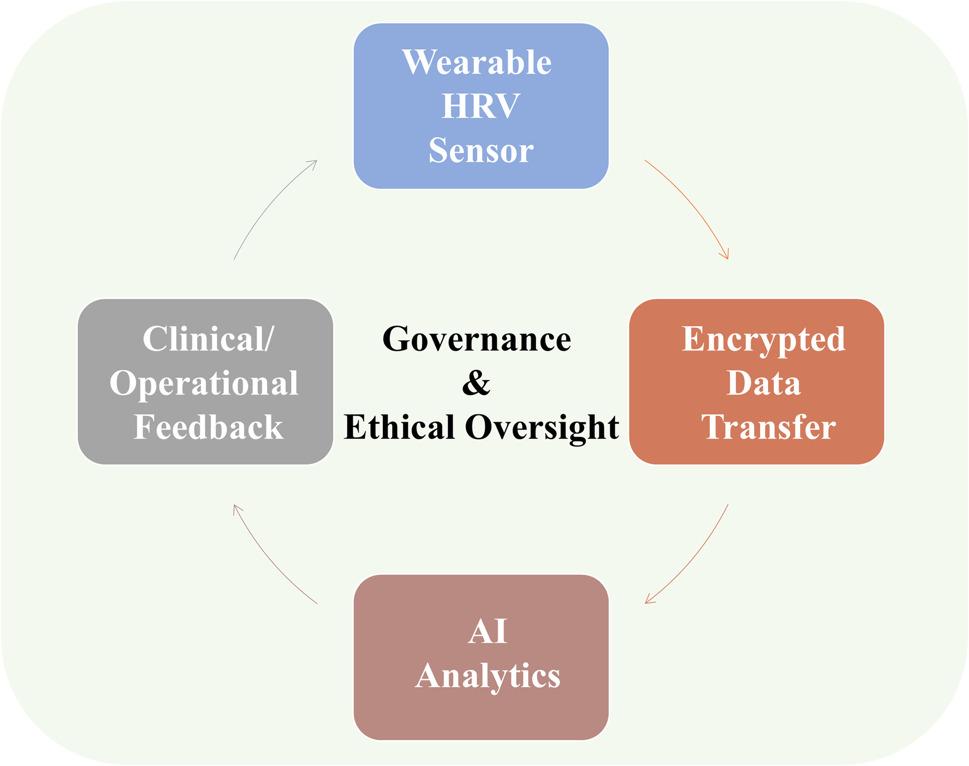
Architecture of data governance and feedback in HRV analytics

In current NATO and U.S. military telemedicine architectures, data from wearable monitors are sent through encrypted medical communication channels comparable to those used in hospital systems, ensuring that patient identifiers and physiological traces cannot be intercepted or altered. Instead of open radio or unsecured Wi-Fi, these devices use protected digital pathways, essentially medical VPNs, so that data packets reach field servers or telemedicine hubs safely and without corruption (Fig. 3) [93].

Behind the front line, these medical coordination hubs function like digital intensive-care units: physiological data streams from multiple soldiers are integrated into real-time dashboards within telemedicine platforms such as the Tactical Telemedicine Network or the MHS GENESIS Defense Health Environment. Here, HRV, oxygen saturation, skin temperature, and motion signals are automatically analyzed by AI algorithms trained to recognize early warning signs of collapse or severe stress. When certain thresholds are crossed, such as a rapid fall in HRV combined with a rise in heart rate—automated alerts are generated, prompting the supervising clinician to contact field medics or initiate an evacuation decision [94].

From a medical standpoint, these encrypted pathways and automated alarm systems protect both the integrity of clinical decision-making and the safety of the personnel being monitored. Data manipulation or signal loss could lead to false reassurance or unnecessary alarms; hence, recent guidelines from NATO STO (*TR-HFM-333*,* 2023*) and the U.S. Department of Defense emphasize continuous verification of signal authenticity and time-stamped traceability through secure data logs. The European AI Act (2023) and the FDA’s Digital Health Cybersecurity Guidance (2024) similarly require that all physiological data used for clinical or operational analytics remain tamper-proof and traceable [9].

Ultimately, HRV monitoring within these secure telemedical frameworks ensures that information gathered in the most austere environments can reach medical teams in real time –protected, reliable, and clinically meaningful. This model of secure physiological intelligence transforms HRV from an isolated biomarker into a trusted medical signal that supports triage, early intervention, and coordinated care across the entire chain of survival, from the combat zone to the base hospital.

## Towards a dual-use framework for HRV

The integration of HRV analytics across medical, operational, and technological domains points toward the emergence of a unified dual-use framework, a system in which the same physiological signal serves both clinical care and mission performance.

This framework rests on three pillars: (1) scientific validity and physiological interpretability, (2) secure and interoperable data architecture, and (3) ethical governance ensuring that human monitoring remains protective, not coercive.

Clinically, HRV provides objective, real-time insight into autonomic balance, recovery, and stress tolerance, complementing conventional vital signs and supporting the development of preventive or personalized interventions. Operationally, it functions as an index of readiness, fatigue, and decision-fatigue, guiding rest cycles, task allocation, and safety thresholds. When coupled with AI-based analytics and encrypted telemedicine networks, HRV becomes an actionable component of predictive human-performance medicine—enabling early warnings, adaptive feedback, and cross-sector data translation [95].

A dual-use HRV ecosystem would therefore align hospital, occupational, and defense infrastructures through shared standards: validated sensor accuracy (ECG/PPG equivalence), harmonized HRV metrics (SDNN, RMSSD, HF/LF power), interoperable data formats (HL7 FHIR, IEEE 11073), and secure communication layers consistent with NATO STO and DoD cyber-health protocols. Such integration would allow physiological intelligence collected in the field to inform civilian healthcare and vice versa, creating a continuous learning loop between resilience research and patient recovery.

In practical terms, this framework envisions HRV as a translational bridge, linking critical-care telemetry with operational monitoring, supporting both the soldier on deployment and the patient in rehabilitation. Its success depends on sustained collaboration between clinicians, physiologists, data scientists, and defense-health planners. Establishing standard repositories, standardized validation protocols, and clinician-led oversight boards will ensure that HRV analytics remain scientifically rigorous, secure, and ethically aligned with the fundamental mission of medicine: to protect human life and sustain performance without compromising dignity or trust [90].

## Conclusions

The integration of HRV into both clinical and operational frameworks supports a more unified interpretation of autonomic physiology across human performance and health, while highlighting the need for standardized acquisition, context-aware interpretation, and setting-specific validation. Within military medicine, HRV has evolved from a passive indicator of cardiac risk to an active tool for assessing readiness, resilience, and recovery under conditions of sustained stress. Its adoption across defense environments demonstrates that the same autonomic markers guiding individualized rehabilitation and preventive cardiology can also inform mission planning, fatigue management, and early detection of physiological decompensation. By implementing HRV analytics within secure telemedicine architectures and AI-assisted monitoring systems, modern armed forces are advancing toward a model of predictive operational medicine, one that protects personnel through continuous, data-driven insight rather than reactive intervention. This dual-use perspective underscores HRV’s unique translational value: it connects the precision of clinical telemetry with the exigencies of the battlefield, supporting a continuum of care that extends from intensive care units to deployed field operations. The future of HRV lies in this intersection, where scientific rigor, technological innovation, and ethical standards converge to sustain both human health and operational effectiveness.

## Data Availability

This article is a narrative review and does not include original datasets. All data supporting the findings of this study are available within the cited peer-reviewed literature.
